# Risk factors and prediction model of severe pertussis in infants < 12 months of age in Tianjin, China

**DOI:** 10.1186/s12879-021-07001-x

**Published:** 2022-01-04

**Authors:** Cui Zhang, Yanmei Zong, Zhe Wang, Li Wang, Ying Li, Yuejie Yang

**Affiliations:** 1 Department of Infectious Disease, Tianjin Second People’s Hospital, No. 7 Sudinan Road, Nankai District, Tianjin, 300192 China; 2 Department of Pharmacy, Tianjin Second People’s Hospital, Tianjin, 300192 China

**Keywords:** Pertussis, Infant, Whooping cough, Risk factors

## Abstract

**Background:**

To identify risk factors associated with the prognosis of pertussis in infants (< 12 months).

**Methods:**

A retrospective study on infants hospitalized with pertussis January 2017 to June 2019. The infants were divided into two groups according to the severity of disease: severe pertussis and non-severe pertussis groups. We collected all case data from medical records including socio-demographics, clinical manifestations, and auxiliary examinations. Univariate analysis and Logistic regression were used.

**Results:**

Finally, a total of 84 infants with severe pertussis and 586 infants with non-severe pertussis were admitted. The data of 75% of the cases (severe pertussis group, n = 63; non-severe pertussis group, n = 189) were randomly selected for univariate and multivariate logistic regression analysis. The results showed rural area [P = 0.002, OR = 6.831, 95% CI (2.013–23.175)], hospital stay (days) [P = 0.002, OR = 1.304, 95% CI (1.107–1.536)], fever [P = 0.040, OR = 2.965, 95% CI (1.050–8.375)], cyanosis [P = 0.008, OR = 3.799, 95% CI (1.419–10.174)], pulmonary rales [P = 0.021, OR = 4.022, 95% CI (1.228–13.168)], breathing heavily [P = 0.001, OR = 58.811, 95% CI (5.503–628.507)] and abnormal liver function [P < 0.001, OR = 9.164, 95% CI (2.840–29.565)] were independent risk factors, and higher birth weight [P = 0.006, OR = 0.380, 95% CI (0.191–0.755)] was protective factor for severe pertussis in infants. The sensitivity and specificity of logistic regression model for remaining 25% data of severe group and common group were 76.2% and 81.0%, respectively, and the consistency rate was 79.8%.

**Conclusions:**

The findings indicated risk factor prediction models may be useful for the early identification of severe pertussis in infants.

## Background

Pertussis, also known as whooping cough and the 100-day cough, is an acute respiratory disease and one infectious disease caused by the Gram-negative bacilli *Bordetella pertussis* (*B. pertussisis*) that seriously threaten human life and health [1]. The highest incidence of pertussis has been observed in infants < 1 year of age since the 1990s, with second increase in incidence in adolescents [2]. Based on original descriptions of pertussis, the paroxysmal coughing fits, which may last for weeks or months, were considered so typical of pertussis that they were deemed sufficient for diagnosis [3]. However, more recently, pneumonia has been reported as a common complication of pertussis, particularly with younger patients [4]. In fact, with the success of vaccination, infants who are too young to be fully immunized have become the age group suffering the highest hospitalization rate and mortality from pertussis [5].

Pertussis is a local or regional epidemic disease in developing and developed countries, with frequent outbreaks occurring sporadically at different places around the world [6]. After introduction of pertussis vaccination with either whole-cell pertussis (wP) or acellular pertussis (aP) vaccines, the number of confirmed pertussis cases decreased about 90% with high similarity among countries [7]. Despite high vaccine coverage rates in childhood, a re-increase in notified pertussis cases, also termed pertussis resurgence, has been reported in many countries, a phenomenon which started in the 1980’s for some of them [8]. In most recent modeling, the World Health Organization (WHO) estimated that, globally and annually, 24.1 million cases of pertussis occur in children less than 5 years-old, with the majority in low-income countries, and that 160,700 infants eventually die of complications of pertussis [4]. Thus, prevention of pertussis is an important public health in global.

Karen et al. have reported that severe disease is associated with the manifestation of a number of clinical complications [9]. Severe pertussis can lead to sudden infant death, which is inevitable. At present, the reported mortality rate of pertussis is 1.2–3.0% [10]. There are two situations that lead to this situation. Firstly, part of the reason for this death is that the pathogenesis and mortality mechanism of pertussis are not fully understood. Secondly, some clinicians often do not understand the clinical characteristics and risk factors of severe pertussis, and pay enough attention to these factors. In China, although the incidence rate of pertussis has remained low in recent 20 years, the resurgence occurred in more broadly areas from 2014 [11–13]. Therefore, in this study, we retrospectively collected the clinical data of infants (< 12 months) with pertussis from Tianjin (China) to analyze the risk factors of severe pertussis, which may provide the basis for the prevention and control of pertussis and avoid pertussis reappearance in the future.

## Materials and methods

### Characteristic of the study population

From January 2017 to June 2019, the infants younger than one-years diagnosed as pertussis in Tianjin Second People’s Hospital were retrospectively reviewed. All infants were healthy full-term without any congenital disease. All the case data, including socio-demographics, clinical manifestations, auxiliary examinations were collected from their medical records. The study was approved by the Ethics Committee of Tianjin Second People’s Hospital (No.2019-46). All infants’ parents or guardian of children therein have signed consents.

### Criteria of case selection

The infants with pertussis were divided into two groups according to the severity of disease: severe pertussis group and non-severe pertussis group. The diagnosis of non-severe pertussis and severe pertussis was consistent with the report of Wang et al. [14]. Those who met the clinical diagnostic criteria for pertussis in the "Suggestions for Diagnosis and Treatment of Pertussis in Children in China" and those who were detected positive by *B. pertussisis* culture or polymerase chain reaction (PCR) test were diagnosed as non-severe pertussis [15]. Infants with pertussis who also have the following complications were diagnosed as severe pertussis: such as pneumonia, apnea, leukocytosis, pulmonary hypertension, seizures and encephalopathy [14, 16, 17].

### Statistical analysis

Descriptive epidemiological methods were used to retrospectively analyze and summarize the socio-demographics, clinical manifestations and auxiliary examinations of the children. SPSS 22.0 statistical software was used to analyze all the data. The measurement data of normal distribution was expressed as x ± s, and t test was used for comparison between groups. Median M (Q1, Q3) was used for measurement data of non-normal distribution, and Wilcoxon rank sum test was used for comparison between groups. Enumeration data were presented as percentage (%). Categorical variables were calculated by chi-square test. P < 0.05 was considered statistically significant.

### Established logistic regression model

A risk factor prediction model of the risk factors of severe pertussis was established based on 75% patients’ data, which was performed by multivariate logistic regression. The logistic regression model for prediction of severe pertussis in infants was established based on the factors screened by multivariate logistic regression analysis: Logit (P) = − 5.467 + 1.921X1 − 0.968X2 + 0.265X3 + 1.087X4 + 1.335X5 + 1.392X6 + 4.074X7 + 2.215X8. Then, the remaining 25% of the data from the severe pertussis and non-severe pertussis groups were used to evaluate the effect of the prediction model for pertussis severity.

## Results

### Patients’ enrollment

**Fig. 1:**
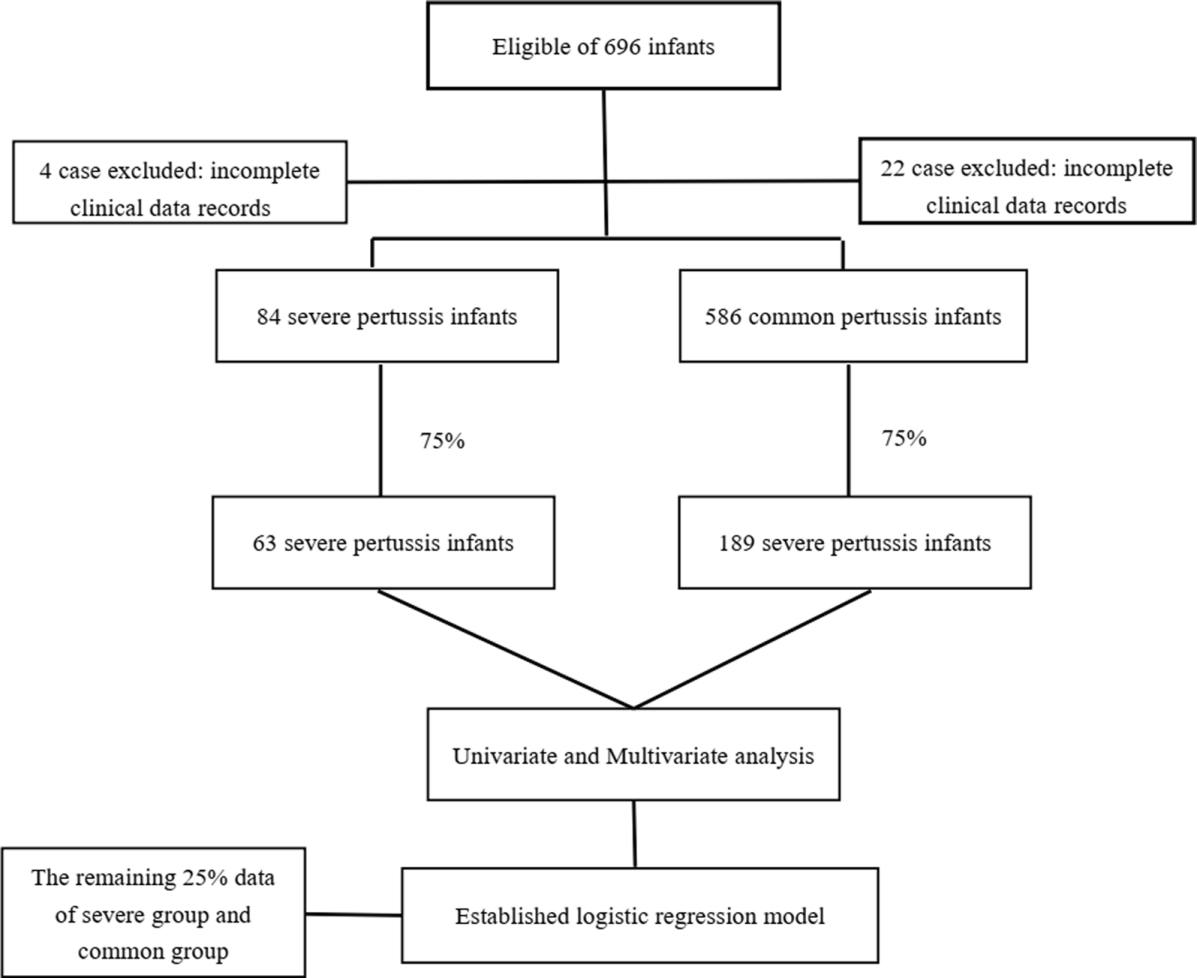
Study cohort creation diagram

A total of 88 laboratory-confirmed infants with severe pertussis were admitted from January 2017 to June 2019, of which 4 were excluded because of incomplete clinical data records, finally 84 cases with severe pertussis were included. During the same period, a total of 608 laboratory-confirmed infants with non-severe pertussis were admitted, of which 22 cases were excluded due to incomplete clinical data records. Among the remaining 586 cases, 252 cases were matched according to the random number table at a ratio of 1:3 as the non-severe pertussis group (Fig. 1).

### Univariate analysis

The data of 75% of the cases (severe pertussis group, n = 63; non-severe pertussis group, n = 189) were randomly selected for univariate analysis, including the socio-demographics (Table 1), clinical manifestations (Table 2) and auxiliary examinations (Table 3) of the children. There were significant differences in age, gender, rural area, artificial feeding, birth and admission weight, vaccination, hospital stay, fever, cyanosis, breathing heavily, decreased heart rate, pulmonary rales, liver function, creatine kinase (CK), creatine kinase-MB (CKMB), serum ferritin, chest CT, co-infection and electroencephalogram (EEG) between severe pertussis group and non-severe pertussis group (all P < 0.05).

**Table 1:** Univariate analysis of socio-demographics between severe group and non-severe pertussis group

Characteristics	Severe pertussis group (n = 63)	Non-severe pertussis (n = 189)	t/χ^2^/Z	P
Age (months)	2 (1, 4)	4 (2, 8)	− 4.478	< 0.001
Gender [(n) %)]				
Boy	38 (60.3)	83 (43.9)	5.093	0.024
Girl	25 (39.7)	106 (56.1)		
Rural area [(n) %)]				
No	10 (15.9)	63 (33.3)	7.001	0.008
Yes	53 (84.1)	126 (66.7)		
Cesarean section [(n) %)]				
No	26 (41.3)	83 (43.9)	0.135	0.714
Yes	37 (58.7)	106 (56.1)		
Premature delivery [(n) %)]				
No	55 (87.3)	178 (94.2)	2.296	0.130
Yes	8 (12.7)	11 (5.8)		
Artificial feeding [(n) %)]				
No	31 (49.2)	130 (68.8)	7.849	0.005
Yes	32 (50.8)	59 (31.2)		
Birth weight (kg)	2.93 ± 0.98	3.34 ± 0.50	3.017	0.004
Admission weight (kg)	6.63 ± 2.71	7.87 ± 2.76	3.086	0.002
Comorbidity [(n) %)]				
No	53 (84.1)	170 (89.9)	1.572	0.210
Yes	10 (15.9)	19 (10.1)		
Contact history [(n) %)]				
No	18 (28.6)	67 (35.4)	1.000	0. 317
Yes	45 (71.4)	122 (64.6)		
Pertussis vaccine (doses)				
0	50 (79.4)	81 (42.9)	25.850	< 0.001
1	5 (7.9)	36 (19.0)		
2	3 (4.8)	15 (7.9)		
≥ 3	5 (7.9)	57 (30.2)		
Hospital stay (days)	13 (11–14)	9 (7–10)	− 7.262	< 0.001

**Table 2:** Univariate analysis of clinical manifestations between severe group and non-severe pertussis group

Characteristics	Severe pertussis group (n = 63)	Non-severe pertussis (n = 189)	t/χ^2^	P
Duration of disease (days)	12.0 (7.0, 14.5)	13.0 (10.0, 15.0)	− 0.857	0.391
Fever				
No	13 (20.6)	90 (47.6)	14.236	< 0.001
Yes	50 (79.4)	99 (52.4)		
Cyanosis [(n) %)]				
No	19 (30.2)	121 (64.0)	21.943	< 0.001
Yes	44 (69.8)	68 (36.0)		
Decreased heart rate [(n) %)]				
No	53 (84.1)	187 (98.9)	19.717	< 0.001
Yes	10 (15.9)	2 (1.1)		
Pulmonary rales [(n) %)]				
No	9 (14.3)	94 (49.7)	25.235	< 0.001
Yes	54 (85.7)	95 (50.3)		
Breathing heavily [(n) %)]				
No	45 (71.4)	188 (99.5)	53.299	< 0.001
Yes	18 (28.6)	1 (0.5)

**Table 3:** Univariate analysis of auxiliary examination between severe group and non-severe pertussis group

Characteristics	Severe pertussis group (n = 63)	Non-severe pertussis (n = 189)	t/χ^2^/Z	P
Liver function [(n) %)]				
Normal	44 (69.8)	169 (89.4)	13.843	< 0.001
Abnormal	19 (30.2)	20 (10.6)		
WBC (× 10^9^/L)	22.71 (15.77, 32.69)	19.77 (15.20, 26.00)	− 1.575	0.115
Lymphocytosis (× 10^9^/L)	15.91 (9.93, 24.14)	13.39 (9.93, 18.60)	− 1.267	0.205
HGB (g/L)	115.0 (106.0, 125.0)	116.0 (109.0, 123.0)	− 0.167	0.868
PLT (× 10^9^/L)	523.0 (415.0, 620.0)	476.0 (371.0, 605.0)	− 1.762	0.078
LDH (U/L)	240.0 (213.0, 299.0)	245.0 (213.0, 274.0)	− 0.670	0.503
AST (U/L)	54.0 (45.0, 67.0)	54.0 (45.0, 69.0)	− 0.526	0.599
CK (U/L)	77.0 (49.0, 110.0)	98.0 (67.0, 138.0)	− 2.295	0.022
CK-MB (U/L)	38.75 (29.80, 50.70)	33.80 (26.60, 43.10)	− 2.072	0.038
C3 (g/L)	0.87 (0.76, 1.03)	0.86 (0.77, 1.01)	− 0.008	0.993
C4 (g/L)	0.19 (0.13, 0.27)	0.18 (0.14, 0.23)	− 0.788	0.431
IgG (g/L)	4.00 (2.60, 5.50)	3.90 (2.10, 5.70)	− 0.764	0.445
IgA (g/L)	0.15 (0.04, 0.34)	0.14 (0.04, 0.28)	− 0.274	0.784
IgM (g/L)	0.62 (0.44, 0.85)	0.66 (0.44, 0.90)	− 0.544	0.586
Serum ferritin (μg/L)	106.0 (56.0, 235.0)	53.0 (29.0, 109.0)	− 3.706	< 0.001
Blood sugar (mmol/L)	5.27 ± 0.90	5.25 ± 0.72	0.250	0.803
CD3 (/L)	10,982.0 (6512.0, 17,082.0)	9156.5 (6206.5, 12,793.5)	− 1.661	0.097
CD4 (/L)	6145.5 (3781.0, 10,593.5)	5402.5 (3774.5, 7742.5)	− 1.641	0.101
CD8 (/L)	4140.0 (1860.0, 6258.0)	3160.0 (2105.0, 4624.5)	− 1.912	0.056
Co-infection [(n) %)]				
No	34 (54.0)	129 (68.3)	4.221	0.040
Yes	29 (46.0)	60 (31.7)		
Chest CT [(n) %)]				
Normal	5 (7.9)	49 (25.9)	22.776	< 0.001
Pneumonia	41 (65.1)	82 (43.4)		
Pulmonary consolidation/atelectasis	10 (15.9)	10 (5.3)		
Others	7 (11.1)	48 (25.4)		
EEG [(n) %)]				
Normal	57 (90.5)	187 (98.9)	8.434	0.004
Abnormal	6 (9.5)	2 (1.1)		

*WBC* white blood cell count, *HGB* hemoglobin, *PLT* platelet, *LDH* lactate dehydrogenase, *AST* aspartate aminotransferase, *CK* creatine kinase, *CKMB* creatine kinase-MB, *EEG* electroencephalogram

### Multivariate logistic regression analysis

Significant factors (P < 0.05) in univariate analysis were analyzed by logistic regression. Multivariate logistic regression analysis showed that sources from rural area [P = 0.002, OR = 6.831, 95% CI (2.013–23.175)], longer hospital stay (days) [P = 0.002, OR = 1.304, 95% CI (1.107–1.536)], occurrence of fever [P = 0.040, OR = 2.965, 95% CI (1.050–8.375)], cyanosis [P = 0.008, OR = 3.799, 95% CI (1.419–10.174)], pulmonary rales [P = 0.021, OR = 4.022, 95% CI (1.228–13.168)], breathing heavily [P = 0.001, OR = 58.811, 95% CI (5.503–628.507)] and abnormal liver function [P < 0.001, OR = 9.164, 95% CI (2.840–29.565)] were independent risk factors for severe pertussis (Table 4). And, birth weight [P = 0.006, OR = 0.380, 95% CI (0.191–0.755)] was protective factor in infants (Table 4), that was the higher the birth weight, the lower the risk of severe pertussis.

**Table 4:** Multivariate logistic regression analysis of risk factors for infants with severe pertussis

Variable	B	S.E	Wald	P	OR	95% CI	
						Lower limit	Upper limit
Constant term	− 5.467	1.743	9.842	0.002	0.004		
Rural area	1.921	0.623	9.504	0.002	6.831	2.013	23.175
Birth weight	− 0.968	0.351	7.615	0.006	0.380	0.191	0.755
Hospital stay	0.265	0.084	10.077	0.002	1.304	1.107	1.536
Fever	1.087	0.530	4.208	0.040	2.965	1.050	8.375
Cyanosis	1.335	0.503	7.054	0.008	3.799	1.419	10.174
Pulmonary rales	1.392	0.605	5.289	0.021	4.022	1.228	13.168
Breathing heavily	4.074	1.209	11.363	0.001	58.811	5.503	628.507
Liver function	2.215	0.598	13.740	< 0.001	9.164	2.840	29.565

*S.E.* standard error, *CI* confidence interval, *OR* odds ratio

### Logistic regression model for prediction of severe pertussis

The rural area (Yes, 1; No 0), higher birth weight (kg), hospital stay (days), fever (Yes, 1; No 0), cyanosis (Yes, 1; No 0), pulmonary rales (Yes, 1; No 0), shortness of breath (Yes, 1; No 0), liver function (abnormal, 1; normal, 0) screened by multivariate logistic regression analysis were used to confirm the Logit (P), and the result showed P cut-off value was 0.3165. The P cut-off value referred to the P-value when the sum of the predicted sensitivity and specificity was maximum. Thus, if the P value was greater than 0.3165, it predicted that the case was likely to progress to severe pertussis; if the P value was less than 0.3165, it predicted that the case was regarded as non-severe pertussis. The remaining 25% data of severe group and non-severe pertussis group were brought into the logistic regression prediction model to test the effect of model. The results showed that the sensitivity and specificity of logistic regression model were 76.2% and 81.0%, respectively, and the consistent rate was 79.8% (Table 5).

**Table 5:** Prediction effect of logistic regression model

Logistic regression model	Diagnosis result		Sensitivity (%)	Specificity (%)	Consistent rate (%)
	Yes	No			
Yes	16	12	76.2	81.0	79.8
No	5	51			
Total	21	63			

## Discussion

In recent years, the incidence of pertussis has resurgence in many countries, such as Australia, Canada, Netherlands and USA [18, 19]. Meanwhile, the incidence rate of pertussis is also rebounding in China. According to China Center for Disease Control and Prevention (CDC), the incidence rate of pertussis in China has dropped from 0.362/10 per million people to 0.1246/10 per million from 2004 to 2013, but increased to about 0.2/10 million in 2007 and 2011 [12, 20]. In 2015, 6658 cases of whooping cough were reported in China, which was nearly double the number reported in 2014 (3408 cases) [11, 20]. Furthermore, severe pertussis and death due to pertussis have gradually increased. Despite the ongoing impact on the health system of this severe yet preventable infection, population-based data in critically ill infants with severe pertussis are limited. The aim of this study was to explore specific risk factors leading to severe pertussis.

Pertussis caused by infection with *B. pertussisis* predominantly seen in infants, some occurrence of complications could lead to severe pertussis, such as pneumonia, apnea, leukocytosis, pulmonary hypertension, seizures and encephalopathy [14, 16, 17]. A recent 5-year surveillance study from Switzerland reported an incidence of laboratory confirmed pertussis of 38.8/100,000 in infants less than 12 months old [21]. According to one research, age 3 months is a risk factor for severe pertussis [22]. In our study, the age of severe pertussis patients ranged 1–4 months, which is consistent with the previous study. Our study showed that the age of children in the severe group was lower than that in the non-severe group, and the difference was statistically significant (P < 0.001). The median age was 2 months, indicating that younger age was a risk factor for severe pertussis. Therefore, the protection of children less than 3 months who had not been vaccinated against pertussis has become a hotspot of current research.

Children with pertussis are often young and unable to discharge sputum autonomically, while pertussis bacilli can cause extensive bronchial inflammation, stimulate sustained immune response, promote hypersecretion of airway mucus, block bronchus, and lead to lung imaging changes [23]. One previous study has reported that breathing heavily, white blood cell count (WBC), lymphocytosis, and pulmonary hypertension (pulmonary rales) were commonly observed and significantly associated with infant deaths from pertussis [24]. In this study, through the retrospective analysis of the clinical characteristics of severe pertussis, we found breathing heavily and pulmonary pulmonary rales were independent risk factors for severe pertussis. The WBC and lymphocytosis in severe pertussis cases were generally abnormal, but not necessarily an independent risk factor for severe pertussis, due to in some infants who have immune deficiency or secondary infection, the increase of WBC or lymphocytosis was not significant increased or even decreased.

One study have also found that higher birth weight, independent of preterm birth, has an important effect on the risk of health services use following exposure to the acellular pertussis vaccine at 2 months of age [25]. Children with low birth weight were significantly more likely to have reported pertussis than were normal birth weight children [26]. In this study, we found higher birth weight [P = 0.006, OR = 0.380, 95% CI (0.191–0.755)] was a protective factor in infants, which was similar to the above studies. We firstly reported the longer hospital stay and occurrence of fever, cyanosis, pulmonary rales, breathing heavily and abnormal liver function were independent risk factors for severe pertussis. In addition, we also found sources from rural area was independent risk factor for severe pertussis, which might indicated the region where these children live should be closely monitored to prevent an outbreak of pertussis. Finally, the prediction model of risk factors obtained by logistic regression analysis, which could provide a more accurate theoretical basis for the evaluation of the degree of illness of children with pertussis, and provide a reference for the early identification of severe pertussis.

## Conclusion

The findings indicated the rural area, hospital stay (days), fever, cyanosis, pulmonary rales, breathing heavily and abnormal liver function were independent risk factors for severe pertussis, and higher birth weight was protective factor for severe pertussis in infants. Risk factor prediction models may be useful for the early identification of severe pertussis in infants.

## Data Availability

All data generated or analyzed during this study were included in this published article.

## Abbreviations

*B. pertussisis*: *Bordetella pertussis*
wP: Whole-cell pertussis
aP: Acellular pertussis
WHO: World Health Organization
PCR: Polymerase chain reaction
CDC: Control and Prevention
WBC: White blood cell count
CK: Creatine kinase
CKMB: Creatine kinase-MB
EEG: Electroencephalogram
